# Advances in the application of neuroinflammatory molecular imaging in brain malignancies

**DOI:** 10.3389/fimmu.2023.1211900

**Published:** 2023-07-18

**Authors:** Wenxia Dong, Ning Wang, Zhe Qi

**Affiliations:** ^1^ Department of Radiology, The First People’s Hospital of Linping District, Hangzhou, China; ^2^ Department of Medical Imaging, Jining Third People’s Hospital, Jining, Shandong, China; ^3^ Department of Radiology, Zibo Central Hospital, Zibo, Shandong, China

**Keywords:** magnetic resonance imaging, positron emission tomography, neuroinflammation, brain tumor, metastasis

## Abstract

The prevalence of brain cancer has been increasing in recent decades, posing significant healthcare challenges. The introduction of immunotherapies has brought forth notable diagnostic imaging challenges for brain tumors. The tumor microenvironment undergoes substantial changes in induced immunosuppression and immune responses following the development of primary brain tumor and brain metastasis, affecting the progression and metastasis of brain tumors. Consequently, effective and accurate neuroimaging techniques are necessary for clinical practice and monitoring. However, patients with brain tumors might experience radiation-induced necrosis or other neuroinflammation. Currently, positron emission tomography and various magnetic resonance imaging techniques play a crucial role in diagnosing and evaluating brain tumors. Nevertheless, differentiating between brain tumors and necrotic lesions or inflamed tissues remains a significant challenge in the clinical diagnosis of the advancements in immunotherapeutics and precision oncology have underscored the importance of clinically applicable imaging measures for diagnosing and monitoring neuroinflammation. This review summarizes recent advances in neuroimaging methods aimed at enhancing the specificity of brain tumor diagnosis and evaluating inflamed lesions.

## 1 Introduction

The most prevalent brain tumors comprise meningiomas, gliomas—particularly glioblastomas (GBMs)—and intracranial metastases originating from various cancers (1). The treatment approach for intracranial metastases involves a combination of chemoradiotherapy and neurosurgery (2). Meningiomas are predominantly benign and are typically managed by surgical resection (3). GBM represents the primary malignancy in the central nervous system (CNS) and is known for its aggressive nature, with limited benefits derived from advanced therapeutic strategies (4). Therefore, early diagnosis and accurate monitoring through neuroimaging are crucial for managing brain tumors. Neuroimaging is an indispensable aspect of clinical practice, offering valuable insights into brain tumors.

The advancement of computed tomography (CT) and magnetic resonance imaging (MRI) has enabled more precise diagnoses, improved clinical monitoring, and enhanced accuracy in prognostic prediction (1). In particular, neuroimaging has played a crucial role in diagnosing and treating brain tumors. By combining molecular pathology and histopathology, significant progress has been made in classifying various types of brain tumors. Precision neuro-oncology integrates tumor-specific neuroinflammation and distinct protein alterations (5). However, the translation of the precision oncology paradigm to neuro-oncology faces several significant challenges, including the criteria for assessing therapeutic effects and the need for accurate monitoring approaches. Advanced neuroimaging techniques currently offer potential solutions to overcome these challenges. The interactions between brain tumors and immune responses are critical focal points (6, 7). Consequently, current immunotherapeutic strategies have been developed to target specific immune cells or inflammatory mediators within the tumor microenvironment (TME) (8). Neuroinflammation plays a crucial role in the progression of brain tumors. GBM, in particular, is characterized by tissue necrosis accompanied by heightened neuroinflammation. Additionally, immunosuppressive neuroinflammation and induced necrosis also contribute to treatment resistance and poor prognosis (5, 9). However, immunotherapies also affect imaging phenotypes in clinical practice. Therefore, it is essential to gain a better understanding of the inflammatory factors and their associated neuroimaging characteristics.

In this review, the key aspects of neuroimaging in diagnosing and distinguishing brain tumors from inflammatory-associated lesions were emphasized. This information is immensely valuable for gaining a better understanding of imaging characteristics and common patterns that aid in diagnosis. Furthermore, a comprehensive understanding of the inflammatory mechanisms within the TME and their corresponding imaging features facilitates the development of noninvasive prognostic and predictive imaging strategies in clinical practice.

## 2 Current limitations in neuroimaging in the context of brain malignancy

The clinical diagnosis, evaluation, and monitoring of therapeutic effects in patients with brain malignancies heavily rely on neuroimaging techniques. There is a wide range of neuroimaging options available for clinical use. Structural MRI is a useful choice for identifying and classifying tumors and guiding surgical strategies. Additionally, PET imaging is predominantly used to assess tissue metabolism, providing valuable information about cancer cell proliferation and early-stage tumor detection (10). PET is a type of molecular imaging technique that is suitable for detecting specific molecules, such as choline, fluorodeoxyglucose, methionine, and phenylalanine (11, 12). Thus, the critical mechanism of PET-based neuroimaging involves defining and tracing the targeting molecules that are specific or sensitive to immune cells, including microglia and macrophages.

The efficiency of current neuroimaging techniques is widely acknowledged; however, there are still several limitations requiring further improvement. For instance, distinguishing relapsed brain tumors, brain metastasis, or inflammatory and necrotic lesions poses a significant challenge in clinical treatment, as both tumors and metastatic sites exhibit similar intratumoral textures on MRI (13, 14). The application of PET techniques also faces various challenges. The low permeability of biotracers across the blood-brain barrier (BBB) and systemic plasma binding affect the imaging results. Additionally, inherent characteristics of the neuroimaging technique itself impose certain limitations. The most commonly used PET probe for neuroimaging, 2-deoxy-2-18F-fluoro-D-glucose, is based on glucose metabolism (15). Glucose transporters are highly expressed not only in tumor cells but also the inflamed areas (16). Importantly, glucose metabolism plays a crucial role in inflammatory responses, leading to false-positive results in tumor diagnosis. Therefore, in this review, the recent advances in neuroimaging approaches aimed at improving the evaluation of brain tumors and brain metastases combined with neuroinflammatory response information have been discussed. The first radiotracer used for neuroinflammation is based on an 18-kDa protein named translator protein (TSPO), a peripheral benzodiazepine receptor (17). TSPO is a mitochondrial transmembrane protein predominantly located in macrophages and microglia (18). Accumulative evidence indicated that TSPO up-regulation can be observed in pro-inflammatory and immunosuppressive conditions. Previous studies have shown that TSPO expression can be up-regulated in response to neuroinflammation and tumor malignancy (19). Additionally, TSPO can also be found in neoplastic glioma cells. Consequently, over 13 unique TSPO radiotracers have been developed for use in malignant brain pathologies, enhancing tumor-to-background brain signals (20). Furthermore, TSPO-based PET imaging shows promise in delineating clinically important neuroinflammatory tumor features (21). Therefore, the establishment of novel neuroimage techniques is urgently required for patients with brain tumors.

## 3 Brain tumors as neuroinflammatory diseases: tumor immune microenvironment

Neuroinflammation plays a crucial role in brain TME, resulting in carcinogenesis and tumor progression. In particular, primary brain malignancies, such as GBMs, are characterized by significant immunosuppressive neuroinflammation and necrosis, resulting in resistance to chemotherapy and poor clinical outcomes (22). Tumor cells produce and secrete various immunomodulatory substances, including interleukin (IL)-10, IL-1β, galectin-1, and transforming growth factor-β, which regulate the behavior of infiltrating immune cells and establish a pro-tumoral microenvironment (23, 24). Among the components of the pro-tumoral microenvironment, tumor-associated macrophages (TAMs) are particularly prominent (6, 25). Neuroinflammation induces immunosuppressive changes and further enhances the proliferation, migration, and therapeutic resistance of tumor cells (26).

### 3.1 Immune component of the TME in CNS malignancies

Under normal conditions, the CNS is protected by the BBB, which renders it immune-privileged and shields it from the influence of systemic inflammation. However, immune surveillance and inflammatory regulation in the CNS are mediated by resident immune cells and infiltrating peripheral cells. The microenvironment within brain tumors comprises a diverse array of cellular and immune components, which significantly affect the growth, drug resistance, and recurrence of primary and metastatic brain tumors (27, 28).

First and foremost, it has been reported that astrocytes can be immediately infiltrated around brain tumors, exhibiting varying morphology and cellular functions (29). Active astrocytes, notably, have the ability to promote the invasive capacity and drug resistance of tumor cells, thereby enhancing tumor growth and survival, while suppressing the therapeutic efficiency in clinical treatment (30). Conversely, several studies have also reported the anti-tumor function of astrocytes. It has been demonstrated that specific astrocyte-derived exosomes comprising micro-ribonucleic acid (RNA) can significantly inhibit tumor growth (31). Additionally, microglia, macrophages, endothelial cells, and pericytes play key roles in tumor growth and neuroinflammation, potentially serving as clinical imaging features (32). Accumulative evidence illustrates the critical roles of microglia and macrophages in tumor malignancy, accounting for more than 30% of the cell populations (33). Microglia derived from the yolk sac precursor cells during embryonic development are resident phagocytic cells, while macrophages are derived from the bone marrow-derived monocytes (BMDMs) and migrate into the brain parenchyma. Lineage-tracing experiments in brain metastases have demonstrated the infiltration of microglia and BMDMs in such malignancies. Collectively, microglia and macrophages are widely considered to be associated with tumor malignancy, referred to as TAMs (34).

The tumor vasculature primarily comprises endothelial cells, playing a critical role in tumor growth and facilitating immune reactions within the TME through the release of various neuroimmune substances, including IL-1β, IL-10, and granulocyte-macrophage colony-stimulating factor (GM-CSF) (35, 36). Additionally, pericytes are localized around the vascular vessels and are responsible for tumor vascularization and maintaining BBB integrity (37). The tumor vasculature comprises established vasculature and neo-vasculature, with the latter being significantly enhanced under the regulation of local hypoxia, metabolic demands, and vascular endothelial growth factor stimulation (38). Furthermore, the tumor vasculature exhibits abnormalities in integrity and function. Previous studies have shown a higher prevalence of dysfunctional vasculature within the tumor region, while the peritumoral vasculature resembles normal blood vessels. Following the intravenous administration of a gadolinium-based contrast agent (GBCA), the GBCA enters the extracellular space in the brain parenchyma through this abnormal vasculature, resulting in hyperintensity on T1-weighted magnetic resonance (MR) images (39). However, tumor cells might still be present in the non-enhancing regions around the areas of enhancement. Therefore, the presence or absence of tumor cells is not directly associated with the compromised BBB, increasing the difficulty of clinical diagnosis.

### 3.2 The interactions between the immune responses and brain tumors

The CNS possesses a unique immune monitoring system that involves meningeal or brain parenchymal lymphatic vessels and immune surveillance. TAMs can be classified into pro-inflammatory (M1) or anti-inflammatory (M2) types based on their cellular function and morphological features, which are associated with various processes such as the inflammatory cascade, immune activation, angiogenesis, tissue remodeling, and tumor survival. This classification framework allows for a better understanding of the multifaceted roles of TAMs (40). M1 TAMs are responsible for pathogen recognition and tumor killing. On the contrary, M2 TAMs primarily regulate inflammatory reactions and establish a favorable TME through the release of anti-inflammatory cytokines, thereby promoting tumor survival and growth (41).

Furthermore, the dysregulated adaptive immune reactions within the TME also contribute to tumor growth. The function and prognostic predictive value of adaptive immune cells in brain tumors have gained considerable attention. A previous clinical study has shown a correlation between prolonged survival in patients with GBM and the infiltration of the cluster of differentiation CD 8^+^ T lymphocytes, whereas regulatory T cells are associated with worse prognosis in patients (42, 43). The adaptive immune responses are profoundly influenced by immune checkpoints, which regulate the immune response to self-antigens and contribute to tumor control. Accumulating evidence indicates that various tumors, including CNS malignancies and metastases, evade recognition and surveillance by the immune system through immune checkpoint regulation (44).

Evidence has revealed that therapeutic mobilization of innate and adaptive immune responses can be employed to induce immune-mediated tumor cell death. As a result, a wide array of immunotherapeutic strategies has been established, targeting the interaction between tumor cells and immune cells. These strategies include immune checkpoint inhibition, engineered chimeric antigen receptor T cells, oncolytic viral therapies, and vaccines (45, 46). The underlying mechanisms of immune checkpoint inhibition and vaccines involve the activation of T lymphocytes by eliminating inhibitory signals. Additionally, tumor antigens are presented to antigen-presenting cells, such as dendritic cells (47). These cells are then administered to patients with GBMs after being stimulated with tumor antigens, triggering adaptive immune activation. Based on this approach, vaccine-based immunotherapies use tumor-associated antigens to enhance cytotoxic effects through the enhancement of immune reactions. Due to the critical role of immune components within the TME, neuro-inflammation-based imaging techniques may be a potentially effective approach in the diagnosis of patients with brain tumors.

## 4 Novel neuroimaging approaches for evaluating brain tumor recurrence

Despite the current efficiency of existing neuroimaging techniques in brain tumor detection, there is still a huge challenge in distinguishing recurrent brain tumors, solitary brain metastases and inflammatory and necrotic lesions (48). It has been revealed that conventional neuroradiological techniques struggled to distinguish recurrent tumors and inflammatory alterations, due to their similarly-showing imaging in MRI. Therefore, a better understanding of novel approaches to diagnose recurrent brain tumors may present great clinical potential in the follow-up of patients after anti-tumor therapies. With the rapid advances in neuroimaging, the direct labelling of critical immune cells has emerged as a potential option for neuroinflammation imaging with clinical relevance. Given that TAMs are considered the predominant immune cells within the brain TME, significant efforts have been made to label TAMs based on accumulating evidence. Furthermore, MRI plays an essential role in clinical assessment and monitoring, making novel approaches using MRI for neuroinflammation imaging likely to be applicable in clinical practice. In conventional MRI, GBCAs are commonly used to examine contrast dynamics and identify CNS malignancies. However, it is noteworthy that ultrasmall superparamagnetic iron oxide (USPIO) nanoparticles possess a unique capability to detect neuroinflammation by being taken up by immune cells (49). One such USPIO nanoparticle is ferumoxytol, which has been extensively studied in patients with brain tumors (49). Previous clinical studies have indicated that ferumoxytol exhibits a preferential accumulation in inflamed sites (50). Similarly, an experimental study revealed that ferumoxytol is predominantly observed in activated astrocytes, microglia, and macrophages but not in tumor cells. This underscores the potential of ferumoxytol-enhanced MRI for neuroinflammation imaging in patients with brain tumors (51). Currently, ferumoxytol has received Food and Drug Administration approval for clinical use. In cases where the administration of GBCA is restricted due to clinical conditions such as GBCA allergy or renal failure, ferumoxytol is considered an alternative contrast agent (52). Additionally, ferumoxytol has demonstrated potential advantages over GBCA in the assessment of neuroinflammation. Its plasma half-life is approximately 14-21 h, allowing delayed localization within TAMs or the neovascular space (53). This prolonged intravascular signal persistence contributes to the observed parenchymal signal during delayed imaging (54). A prospective pilot study involving patients with gliomas revealed that delayed ferumoxytol imaging captures TAM signals within the TME. Furthermore, there is a positive correlation between susceptibility and relaxation and the number of macrophages (54).

The interpretation of T1-weighted ferumoxytol contrast-enhanced MRI depends on clinical conditions (55). During the diagnostic phase, similar imaging characteristics can be observed in primary or metastatic brain tumors using either GBCA or ferumoxytol-enhanced MRI. However, after chemoradiotherapies, these enhancing images might differ. Specific immunotherapies can induce neuroinflammation, leading to gadolinium enhancement and T2 hyperintensity on MRI. Although the enhanced signals resemble those of solid tumors, they subsequently undergo spontaneous regression, known as pseudoprogression. Psuedoprogression can be observed in approximately 30% of patients with GBMs after chemotherapy or radiotherapy. Given the underlying mechanisms of immunotherapies, neuroinflammation induced by immunotherapies is more likely to be detected than that induced by chemoradiotherapies. Therefore, distinguishing pseudoprogression from actual tumor growth becomes crucial. Additionally, iron nanoparticle imaging requires specific attention. The prolonged clearance of iron nanoparticles should be given careful consideration in clinical practice (56). A previous study has revealed an increased level of methionine in metastatic tumor tissues (57, 58). Furthermore, elevated levels of the neuroinflammatory marker PBR-TSPO can be observed in necrotic sites and quantified using a specific PET biotracer. Therefore, PET biotracers based on ^11^Carbon (C)-methionine have been used in patients with metastatic brain tumors to accurately diagnose tumor recurrence (59, 60). In one clinical trial, ^11^C-methionine-based PET biotracers correctly diagnosed tumor recurrence, as confirmed by pathological examination, in seven patients. In comparison, ^11^C-PBR28-based PET biotracers only identified three lesions, highlighting the reliability of ^11^C-methionine-based biotracers for tumor recurrence detection. Thus, ^11^C-methionine is considered a reliable marker for tumor recurrence compared with ^11^C-PBR28 PET (61). PET imaging provides significant benefits in advanced photon techniques, particularly in treatment planning and the sparing of normal tissue for skull base meningioma using advanced photons and protons. Additionally, incorporating ^68^Gallium-DOTATOC-PET information has a substantial impact on target volumes (62). Moreover, the combination of Multiparametric ^18^F-FET PET/MRI improves the therapeutic effectiveness by distinguishing between tumor progression and therapy-related alterations (63). According to another clinical study, combined dynamic and static ^18^F-FET PET/CT parameters can be used in differentiating radiation necrosis from recurrent tumor after cyberknife robotic radiosurgery (64). Effective imaging techniques are still required to be explored in future studies.

## 5 Neuroimaging approaches for evaluating brain tumors and radiation-induced necrosis

Currently, MRI (**Table 1**), PET, and single-photon emission CT (SPECT) are the predominant imaging techniques for the noninvasive assessment of neuroinflammation (87). Among them, SPECT can detect gamma rays emitted by radioactive isotopes used in clinical imaging. By employing various radiotracers, SPECT enables precise evaluation of metabolic and molecular processes, such as glucose utilization, nucleoside and amino acid transporter expression, and protein and deoxyribonucleic acid (DNA) synthesis (88). SPECT has been extensively used to investigate the molecular neurodegeneration mechanisms in drug addiction and to enhance therapeutic strategies with minimal adverse effects. Furthermore, SPECT plays a valuable role in neuroscience research, particularly in neurodegeneration and neuro-oncology (89).

**Table 1:** Magnetic resonance imaging (MRI)-based neuroimaging applied in brain tumors.

Imaging approach	Application	Image features	Author	Year
Proton magnetic resonance (MR) spectroscopy	To differentiate tumor recurrence from radiation necrosis	Increased lactate/creatine (Cr) ratio and decreased choline (Cho)-containing compounds/Cr ratio in necrosis or all the major metabolites were completely diminished.	Kamada et al.	1997 (65)
Two-dimensional (2D) chemical shift imaging MR spectroscopy	To differentiate tumor recurrence from radiation necrosis	Diagnostic spectra can be obtained in 97% of the patients. The Cho/N-acetylaspartate (NAA) and Cho/Cr ratios are the best numeric discriminators.	Weybright et al.	2005 (66)
T2-weighted dynamic susceptibility-weighted contrast material-enhanced (DSC) MRI	To differentiate tumor recurrence from radiation necrosis	Significantly higher relative peak height (rPH) and relative cerebral blood volume (rCBV) and lower relative percentage signal recovery (PSR) values in patients with recurrent glioblastomas (GBMs) than in patients with radiation necrosis.	Barajas et al.	2009 (67)
Proton MR spectroscopy	To differentiate tumor recurrence from radiation necrosis	Increasing Cho levels in patients with radiation necrosis.	Nakajima et al.	2009 (68)
MR spectroscopy and MR perfusion	To differentiate tumor recurrence from radiation necrosis	Cho/NAA and Cho/Cr ratios and rCBV more accurately differentiate between necrosis and recurrent tumors.	Chuang et al.	2016 (69)
Diffusion tensor imaging (DTI) and calculation of the apparent diffusion coefficient (ADC)	To differentiate tumors from radiation abscesses	Hyperintense signal changes in abscesses. The combination of DTI and dynamic susceptibility contrast perfusion-weighted imaging improves the differentiation between tumors and brain infections.	Bink et al.	2005 (70)
Calculation of the ADC	To differentiate tumors from radiation abscesses	The accuracy of ADC ratios in discriminating brain abscesses from cystic or necrotic neoplasms is high and can be further improved with the use of T2 rim characteristics.	Fertikh et al.	2007 (71)
MR spectroscopic imaging	To differentiate tumors from radiation abscesses	Metabolite ratios and maximum Cho/Cho-n, Cho/Cr, and Cho/NAA ratios of the contrast-enhancing rim could differentiate abscesses from brain tumors.	Lai et al.	2008 (72)
MR spectroscopy	To differentiate high-grade gliomas from metastases	Intratumoral Cr is suggestive of a glioma. The absence of Cr indicates metastasis.	Ishimaru et al.	2001 (73)
Phase difference enhanced imaging (PADRE) in MRI	To differentiate high-grade glioma from metastases	Evaluation of peritumoral areas on color PADRE helps differentiate GBMs from metastases	Doishita et al.	2018 (74)
Computational-aided quantitative image analysis (T2-weighted/susceptibility-weighted/contrast-enhanced T1-weighted MRI)	To differentiate high-grade gliomas from metastases	Computational-aided quantitative analysis of MRI improves diagnostic accuracy while differentiating GBM from metastases.	Petrujkić et al.	2019 (75)
Resonance imaging texture analysis	To differentiate high-grade gliomas from metastases	The peritumoral edema was higher than the edema surrounding the metastatic tumor.	Skogen et al.	2019 (76)
Combining arterial spin labelling perfusion (ASL)- and DTI-derived metrics	To differentiate high-grade gliomas from metastases	A combination of ASL- and DTI-derived metrics of the peritumoral part helps differentiate between GBMs and brain metastasis	Abdel et al.	2019 (77)
Multilayer perceptron (MLP) models with non-enhancing T2 hyperintense regions	To differentiate high-grade gliomas from metastases	A trained multi-class MLP model using parameters from preoperative MR images could help differentiate between GBMs, brain metastases, and central nervous system lymphomas.	Swinburne et al.	2019 (78)
Calculation of the ADC	To differentiate high-grade gliomas from metastases	Higher homogeneity and the inverse difference moment in GBMs compared with metastases	Zhang et al.	2019 (79)
Evaluate the rCBV gradient in the peritumoral brain zone (PBZ)	To differentiate high-grade gliomas from metastases	The rCBV gradient derived from DSC MRI in the PBZ is an efficient parameter to differentiate GBMs from brain metastases.	She et al.	2019 (80)
Machine learning method based on texture parameters in MRI	To differentiate high-grade gliomas from metastases	Based on the texture parameters in MRI, the performance of the machine learning method was superior to that of the univariate method while differentiating GBMs from brain metastases.	Tateishi et al.	2020 (81)
2D texture features extracted from MRI	To differentiate high-grade gliomas from metastases	High accuracy employing a set of 2D texture features to discriminate between GBMs and brain metastases.	Ortiz-Ramón et al.	2020 (82)
Three-dimensional T1-weighted (3DT1) MR images with the machine learning classifier	To differentiate high-grade gliomas from metastasis	The proposed diagnostic support system based on radiomics features extracted from post-contrast 3DT1 MR images helps differentiate solitary metastases from GBMs.	de Causans et al.	2021 (83)
MR spectroscopy	To differentiate high-grade gliomas from metastases	No difference in the ADC values and ratios, as well as standard deviation values and ratios between GBMs and brain metastases.	Beig Zali et al.	2021 (84)
A deep learning-based model based on MRI	To differentiate high-grade gliomas from metastases	An efficient deep learning-based model was established and validated using MR images.	Shin et al.	2021 (85)
MRI-based machine learning decision	Identification of medulloblastoma subgroups	MRI-based machine learning helps identify clinically relevant molecular pediatric medulloblastoma subgroups	Zhang et al.	2022 (86)

However, conventional neuroradiological techniques have several limitations. The increased contrast enhancement observed in MRI could lead to difficulty in distinguishing between radiation necrosis and tumor recurrence, as the lesions appear similar. Currently, radiotherapy and stereotactic radiosurgery are commonly used for treating brain metastasis. However, the incidence of radiation-induced necrosis poses a significant challenge in clinically differentiating between metastasis and radiation-induced necrosis. A previous study has demonstrated that conventional MRI has a specificity of 75% and a sensitivity of only 44% in distinguishing between tumors and inflamed necrotic tissue (90, 91).

Neuroinflammation and necrosis are prominent side effects of radiotherapies for treating brain malignancies. A previous retrospective study reported that the rate of radiation-induced necrosis in patients with GBM ranged from 2.5% to 30% (92, 93). This necrotic tissue disrupts the vasculature and perivascular parenchyma, resulting in an abundance of neuroinflammation. The compromised BBB facilitates peripheral immune cell infiltration and brain edema, thereby amplifying the inflammatory response initiated by activated and infiltrated immune cells. Currently, proton MR spectroscopic imaging is employed to assess cellular metabolism by detecting the distribution of proton metabolites, including creatine, lactate, lipid, and especially choline, which is particularly elevated in cell populations exhibiting enhanced cell proliferation. This advanced MRI technique allows for a detailed evaluation of tissue biochemical composition and blood perfusion, providing valuable information for distinguishing solid tumor tissues and necrotic regions. In a previous clinical trial involving 29 consecutive patients, the feasibility and utility of two-dimensional (2D) chemical shift imaging MR spectroscopy were investigated, demonstrating that increased ratios of choline content can differentiate brain tumors from necrotic tissues with an accuracy of up to 97% (66). The choline/N-acetylaspartate and choline/phosphocreatine ratios are particularly effective discriminators in necrotic lesions. However, it is important to note that a transient increase in choline levels can be observed in patients with radiation-induced necrosis, resulting in a false-positive diagnosis of tumor recurrence when using proton MR spectroscopy (68). Another study focusing on patients undergoing high-dose radiotherapy revealed that elevated lactate/creatine ratios and decreased metabolites are more commonly observed in patients with radiation necrosis than in those with recurrent GBM (65).

A retrospective study revealed significant differences in the mean, maximum and minimum relative peak height, and relative cerebral blood volume between patients with GBM and those, as detected by T2-weighted dynamic susceptibility-weighted contrast material-enhanced MRI (67). Furthermore, lower recovery values were observed in recurrent GBM compared with radiation necrosis. Additionally, a meta-analysis of 13 studies demonstrated that the detection of choline/N-acetylaspartate and choline/phosphocreatine ratios, along with relative cerebral blood volume (rCBV) using MR spectroscopy and MR perfusion, significantly improves accuracy in diagnosing primary or metastatic brain tumors (69).

PET scan, as a widely applied noninvasive neuroimaging approach, is useful for imaging neuroinflammation (**Table 2**). According to a previous study, PET scans exhibit a specificity of 69% and a sensitivity of 92% in differentiating between tumor recurrence and radiation necrosis, surpassing nuclear MR spectroscopy for choline/N-acetylaspartate and choline/creatine ratios at various thresholds (98). Another experimental study conducted on orthotopic GBM rat models demonstrated the excellent ability of PET with ^18^Fluorine (F)-fluorodeoxyglucose and ^18^F-fluoroethyltyrosine to distinguish primary GBMs from necrosis (96). Furthermore, a meta-analysis of six studies suggested that ^11^C-choline PET is the most accurate diagnostic approach for distinguishing tumor relapse from necrosis in patients with glioma (97). Additionally, a retrospective study with long-term follow-up revealed that ^11^C-choline PET/CT outperform MRI and ^18^F-fluorodeoxyglucose in evaluating tumor recurrence or radiation-induced necrosis (94). Furthermore, a previous clinical study involving 50 patients demonstrated that ^11^C-methionine PET outperforms ^11^C-choline and ^18^F-fluorodeoxyglucose PET in distinguishing primary GBMs from necrosis (95). Additionally, the L-type amino acid transporter 1 tumor-specific PET tracer, ^18^F-2-fluoroethyl-L-phenylalanine, is also a superior option compared with ^18^F-fluorodeoxyglucose PET in clinical differentiation, as it exhibits low sensitivity to neuroinflammation. However, the high rate of false-positive and false-negative results in PET scans remains a significant limitation in clinical practice. Therefore, there is an urgent need for improved neuroimaging techniques based on the aforementioned methods to achieve accurate diagnosis and clinical evaluation. Currently, advanced approaches offer substantial benefits in evaluating neuroinflammation through immune substance labelling, assessing BBB integrity *via* contrast agent leakage, and identifying inflammatory consequences combined with phenotypic imaging patterns and imaging genomics.

**Table 2:** Positron emission tomography (PET)-based neuroimaging applied in brain tumors.

Imaging approach	Application	Image features	Author	Year
^11^Carbon (C)-choline PET/computed tomography (CT)	To differentiate recurrent GBMs from radiation necrosis	^11^C-choline PET/CT exhibits higher sensitivity and specificity while differentiating recurrent brain tumors from necrosis compared with ^18^Fluorine (F)-fluorodeoxyglucose (FDG) PET/CT and MRI.	Tan et al.	2011 (94)
^11^C-methionine PET	To differentiate recurrent GBMs from necrosis	^11^C-methionine PET exhibits higher sensitivity and specificity while differentiating recurrent brain tumors from necrosis compared with ^11^C-choline or ^18^F-FDG PET.	Takenaka et al.	2014 (95)
^18^F-FDG delayed PET	To differentiate GBMs from radiation necrosis	^18^F-fluorodeoxyglucose delayed PET helps differentiate GBMs from radiation necrosis	Bolcaen et al.	2015 (96)
^11^C-choline PET	To differentiate recurrent GBMs from radiation necrosis	^11^C-choline exhibits high diagnostic accuracy while differentiating recurrent tumors from radiation-induced necrosis in gliomas.	Gao et al.	2018 (97)
^11^C-methionine PET	To differentiate high-grade gliomas from metastasis	^11^C-methionine was a more reliable recurrent tumor marker than ^11^C-peripheral benzodiazepine receptor 28.	Tran et al.	2020 (61)
^68^Galium-DOTATOC-PET	Helps in treatment planning for skull base meningiomas with advanced photons and protons	The addition of 68Ga-DOTATOC-PET information during treatment planning for skull base meningiomas significantly affects target volumes.	Stade et al.	2018 (62)
PET scan	To differentiate tumor recurrence from radiation necrosis	PET scan had the best sensitivity and specificity, for choline/N-acetylaspartate and choline/creatine ratios across different thresholds.	Menoux et al.	2017 (98)
^18^F-FET PET/MRI	To distinguish between tumor progression and therapy-related alterations	Multiparametric ^18^F-FET PET/MRI improves the therapeutic effectiveness by distinguishing between tumor progression and therapy-related alterations.	Brendel et al.	2022 (63)
^18^F-FET-PET/CT	To differentiate recurrent GBMs from radiation necrosis	Combined dynamic and static 18F-FET PET/CT parameters can be used in differentiating radiation necrosis from recurrent tumor after cyberknife robotic radiosurgery.	Lim et al.	2022 (64)

## 6 Neuroinflammatory molecular-based imaging strategies for brain metastases

Brain metastasis is more prevalent than primary brain tumors, mainly due to the limited therapeutic efficacy against various primary cancer, such as breast, lung, and colorectal cancers. The prognosis for patients with brain metastasis is significantly compromised, underscoring the importance of early detection and accurate diagnosis. The TME plays a crucial role in the development of brain metastasis. Notably, the cancer stem cells (CSCs) are the predominant population involved in mediating metastasis, while neuroinflammation also plays a decisive role. Inflamed regions within the brain parenchyma facilitate the adhesion of peripheral tumor cells to activated endothelial cells, initiating invasion and metastasis. Tissue lesions caused by brain metastasis contribute to the establishment of neuroinflammation, characterized by persistent activation of astrocytes and microglia, increased production and release of pro-inflammatory substances, compromised BBB permeability, and infiltration of immune cells. Consequently, a diverse array of immune cells and inflammatory factors promote the progression of brain metastasis, exhibiting high heterogeneity depending on the origin of the primary malignancy and the specific brain site involved. Similar to GBMs, hypoxia-induced stimulation triggers pro-inflammatory substance expression in CSCs, further promoting a pro-inflammatory phenotype in GBM. These inflammatory factors contribute to tumor growth and metastasis in GBM (99, 100).

Distinguishing between primary brain tumors and brain metastasis using neuroimaging remains a significant challenge in clinical diagnosis. Both primary and metastatic brain malignancies exhibit similar peritumoral hyperintensities and intratumoral texture on MRI. Previous studies have shown limited differences in apparent diffusion coefficient (ADC) measurements between primary brain tumors and brain metastasis. However, several studies have revealed that GBMs exhibit higher homogeneity and inverse difference moment than brain metastasis (79). Regarding peritumoral edema, MRI demonstrates greater heterogeneity of peritumoral edema in GBMs compared with that in metastasis, with high sensitivity and specificity of 80% and 90%, respectively (76). Additionally, the use of 2D texture features extracted from MRI images enables fast and noninvasive discrimination between GBM and brain metastases (82). Machine learning algorithms have gained significant attention in neuroimaging applications to improve the accuracy of clinical diagnosis. Quantitative analysis of MRI using machine learning and deep learning-based models facilitates the differentiation between primary and metastatic malignancies, emphasizing the significance of texture feature analysis (81, 85). Furthermore, texture features extracted from post-contrast three-dimensional T1-weighted MR images, optimized by machine learning classifiers, have demonstrated high diagnostic performance and generalizability in differentiating solitary brain metastasis from GBM with high diagnosis performance and generalizability (83). In pediatric medulloblastoma (MB), radiogenomics combined with MRI-based machine learning offers an opportunity for MB risk stratification. Studies have reported the beneficial use of MRI-based machine learning in identifying four clinically relevant molecular pediatric MB subgroups (86).

A previous study demonstrated that extracting texture features from post-contrast diffusion tensor imaging (DTI) MRI contributes to distinguishing between primary and metastatic brain tumors, offering high performance and generalizability in clinical diagnosis. Moreover, combining arterial spin labelling perfusion and DTI has shown significant clinical value (77). However, no differentiation in ADC values and ratios, as well as standard deviation values and ratios, was observed between GBMs and brain metastasis (84). In computational-aided quantitative analysis of MRI images (T2-weighted/susceptibility-weighted/contrast-enhanced T1-weighted MRI), high accuracy was achieved in differentiating GBMs from metastases, emphasizing the significance of texture features rather than fractal-based features in clinical practice (75). Trained multi-class multilayer perceptron models using non-enhancing T2 hyperintense regions can differentiate glioblastoma, brain metastasis, and CNS lymphoma with modest diagnostic accuracy, resulting in an approximately 19% increase in diagnostic yield (78).

The integration of DTI, neurite orientation dispersion, intracellular or extracellular volume fraction, and metabolite analysis with neuroimaging techniques have promoted accurate clinical discrimination. Furthermore, the rCBV in the peritumoral brain zone (PBZ) distinguishes GBMs from metastases. Moreover, the CBV gradient or color map obtained from phase difference enhanced imaging in the PBZ also serves as an effective approach for distinguishing GBMs from metastasis. Significantly higher rCBV ratios and rCBV gradient were observed in the PBZ of GBMs compared with brain metastasis (80). Intratumoral proton MR spectroscopy reveals high levels of creatine in primary brain malignancies, particularly GBMs, whereas the absence of intratumoral creatine suggests the presence of metastatic brain malignancies (73). Definite lipid signals indicate tumor tissue necrosis, while the absence of lipid signals might rule out metastasis. Currently, the evaluation of peritumoral areas using color phase difference enhanced imaging, a novel phase-related MRI technique, also aids in the differentiation between GBM tumors and metastases (74). The prognosis for patients with brain metastasis is compromised, therefore, early detection and accurate diagnosis of brain metastasis are critical in clinical management.

## 7 Neuroinflammatory molecular imaging to distinguish CNS malignancies from intracranial infections

In neuroimaging, brain abscess manifests as expansile, rim-enhancing masses surrounded by edema, which can resemble necrotic malignant tumors, particularly GBMs (101). Consequently, lesions caused by brain infections, particularly brain abscesses, are often misdiagnosed as brain tumors due to their similar MRI appearance and characteristics. However, brain abscesses and GBMs could cause nonspecific headaches in the absence of fever, focal neurologic deficits, and epileptic seizures (102). Therefore, the development of rapid and accurate diagnostic techniques is necessary to distinguish between brain abscesses and malignancies. Pathological examination reveals that the enhancing rim of GBMs comprises infiltrated tumor cells, whereas the enhancing rim of pyogenic abscesses comprises inflammatory components such as neutrophils, macrophages, and lymphocytes (103, 104). Therefore, the choline/creatine ratio of the rim-enhancing lesion in abscesses is expected to be lower than that in GBMs. During MRI, the ADC and diffusion-weighted imaging provide valuable information for clinical diagnosis. Previous studies have reported that abscesses exhibit hyperintense signal changes on diffusion-weighted imaging, while GBMs demonstrate varying degrees of hyperintense to hypointense signal conversion. Significant differences have been observed in the choline/creatine, choline/N-acetylaspartate, and choline/choline-n ratios within the contrast-enhancing rim, allowing for differentiation between abscess and GBMs (71). Furthermore, combining dynamic susceptibility contrast perfusion-weighted imaging and DTI has shown improved efficacy in distinguishing inflammatory lesions compared with using a single neuroimaging technique. Research has indicated that choline levels in the ring-enhancing portion of abscesses are significantly lower compared with that of brain tumors (70). Moreover, a subsequent clinical study has demonstrated the significant role of MR spectroscopic imaging in discriminating abscesses from brain tumors. Metabolite ratios and maximum choline/choline-n, choline/creatine, and choline/N-acetylaspartate ratios within the contrast-enhancing rim could effectively differentiate abscesses from brain tumors (72).

## 8 Conclusion

Imaging genomics has emerged as a technique for evaluating neuroinflammation, involving the use of novel imaging biomarkers that capture DNA and RNA patterns associated with the biology or immune states of cancers. The imaging features are closely associated with gene expression patterns, mutations, and protein modifications (105). In the context of GBMs, imaging genomics has been employed, and several biomarkers have been established, such as the isocitrate dehydrogenase 1 mutation status and immunoreactivity. A radiogenomic profiling of 60 patients with GBM demonstrated positive correlations between CD68, CSF1 receptor, CD33, CD4, and CBV (106). Thus, imaging genomics holds the potential for bridging the gap between neuroimaging and tumor diagnosis in clinical practice.

In the era of immunotherapy and precision oncology, focusing solely on isolated imaging of brain tumor is not enough to establish predictive biomarkers and define neuroinflammation. Therefore, novel MRI and PET scans based on the tumor-associated neuroinflammation have attracted great attention. Secondary surgery towards recurrent brain tumors always accompanies elevating surgical risk and high therapeutic costs, therefore, it is important for accurate discrimination between tumor recurrence and radiation-induced necrosis. Multi-parametric MRI presents versatile imaging information and is considered an effective and useful imaging approach in clinical diagnosis. Accumulative evidence emphasized the clinical value of the apparent diffusion coefficient, volume transfer constant, and relative cerebral blood volume in the distinction of tumor cancer, radiation-induced necrosis, and other brain diseases in daily neuro-oncological practice. PET scans present a unique function in determining tumor microenvironment, assessing drug delivery, and evaluating therapeutic effects. Although the application of PET scans presents great advantages in clinical use, high economic costs and restricted devices limit its generalization. The development of neuroimaging and the combination of novel MRI contrast agents and PET radiotracers and imaging genomic techniques enable the evaluation of neuroinflammatory components and the improvement of accurate diagnosis and clinical discrimination. Of note, there are still several limitations requiring further improvement in neuroinflammation-based neuroimaging to minimize the false-positive diagnosis of tumor recurrence and misdiagnosis with necrosis or intracranial infection. In conclusion, the efforts on such noninvasive neuroinflammation imaging towards accurate diagnosis and personalized therapeutic efficacy monitoring will help the establishment of precision oncology strategies for patients with brain tumors or other malignancy.

## Author contributions

WD and ZQ designed the study; WD wrote the draft; ZQ and NW revised the manuscript. All authors contributed to the article and approved the submitted version.
